# Cellular Organelle-Related Transcriptomic Profile Abnormalities in Neuronopathic Types of Mucopolysaccharidosis: A Comparison with Other Neurodegenerative Diseases

**DOI:** 10.3390/cimb46030169

**Published:** 2024-03-21

**Authors:** Karolina Wiśniewska, Lidia Gaffke, Magdalena Żabińska, Grzegorz Węgrzyn, Karolina Pierzynowska

**Affiliations:** Department of Molecular Biology, Faculty of Biology, University of Gdansk, Wita Stwosza 59, 80-308 Gdansk, Poland; karolina.wisniewska@phdstud.ug.edu.pl (K.W.); lidia.gaffke@ug.edu.pl (L.G.); magdalena.zabinska@phdstud.ug.edu.pl (M.Ż.); grzegorz.wegrzyn@ug.edu.pl (G.W.)

**Keywords:** mucopolysaccharidosis, neurodegeneration, organelles, organelle-related genes, transcriptomics, Golgi apparatus fragmentation

## Abstract

Mucopolysaccharidoses (MPS) are a group of diseases caused by mutations in genes encoding lysosomal enzymes that catalyze reactions of glycosaminoglycan (GAG) degradation. As a result, GAGs accumulate in lysosomes, impairing the proper functioning of entire cells and tissues. There are 14 types/subtypes of MPS, which are differentiated by the kind(s) of accumulated GAG(s) and the type of a non-functional lysosomal enzyme. Some of these types (severe forms of MPS types I and II, MPS III, and MPS VII) are characterized by extensive central nervous system disorders. The aim of this work was to identify, using transcriptomic methods, organelle-related genes whose expression levels are changed in neuronopathic types of MPS compared to healthy cells while remaining unchanged in non-neuronopathic types of MPS. The study was conducted with fibroblast lines derived from patients with neuronopathic and non-neuronopathic types of MPS and control (healthy) fibroblasts. Transcriptomic analysis has identified genes related to cellular organelles whose expression is altered. Then, using fluorescence and electron microscopy, we assessed the morphology of selected structures. Our analyses indicated that the genes whose expression is affected in neuronopathic MPS are often associated with the structures or functions of the cell nucleus, endoplasmic reticulum, or Golgi apparatus. Electron microscopic studies confirmed disruptions in the structures of these organelles. Special attention was paid to up-regulated genes, such as *PDIA3* and *MFGE8*, and down-regulated genes, such as *ARL6IP6*, *ABHD5*, *PDE4DIP*, *YIPF5*, and *CLDN11*. Of particular interest is also the *GM130* (*GOLGA2*) gene, which encodes golgin A2, which revealed an increased expression in neuronopathic MPS types. We propose to consider the levels of mRNAs of these genes as candidates for biomarkers of neurodegeneration in MPS. These genes may also become potential targets for therapies under development for neurological disorders associated with MPS and candidates for markers of the effectiveness of these therapies. Although fibroblasts rather than nerve cells were used in this study, it is worth noting that potential genetic markers characteristic solely of neurons would be impractical in testing patients, contrary to somatic cells that can be relatively easily obtained from assessed persons.

## 1. Introduction

Mucopolysaccharidoses (MPS) are a group of diseases caused by mutations in genes encoding lysosomal enzymes that carry out degradation reactions of glycosaminoglycans (GAGs); thus, these compounds accumulate in lysosomes, impairing the proper functioning of not only lysosomes but also whole cells and tissues [1]. This leads to a number of symptoms, such as short stature, facial dysmorphism, chronic joint pain, organomegaly, or sensory problems [2]. The life expectancy of patients with MPS ranges from 10 to 20 years, depending on the severity of symptoms [2,3].

To date, 12–14 types/subtypes (depending on the MPS definition) of this disease have been described, which are differentiated by the type of GAG accumulated and the non-functional lysosomal enzyme. The characteristics of each type of MPS are shown in Table 1 [4]. Some of the MPS types/subtypes (severe cases of MPS I and MPS II, and all of MPS III and MPS VII) are characterized by neurological abnormalities. These symptoms can result from either (i) direct accumulation of GAGs in nerve cells and resulting damage to the central nervous system (CNS) or (ii) spinal cord compression or hydrocephalus that leads to brain damage. This results in aggression, sleep problems, hearing loss, speech difficulties, and personality changes in patients [5,6]. Patients lose previously acquired cognitive-motor skills and begin to regress, requiring round-the-clock care [7].

Therapies such as hematopoietic stem cell transplantation (HSCT) and enzyme replacement therapy (ERT) are used to alleviate symptoms and improve quality of life for patients with MPS. HSCT is a procedure in which hematopoietic cells (usually bone marrow cells) are transplanted into the patient’s organism to replace abnormal cells (in this case, cells with a missing enzyme). ERT, on the other hand, involves delivering the recombinant enzyme that is missing in the patient’s body. The effectiveness of both of these therapies depends on a number of factors, including the age of the patient, the type of MPS, existing symptoms of the disease, and, in the case of HSCT, also the status of the donor (carrying or not having the disease) and the source of the HSCT tissue. There is also a wide variety of responses to therapy in different tissues of the body. In highly vascularized organs such as the liver and spleen, there is a reduction in GAG levels and an improvement in organ function and size. However, both the transplanted stem cells and the administered enzyme have difficulty reaching tissues such as bone, cartilage, and heart valves. In addition, low penetration through the blood-brain barrier (BBB) and ineffective delivery to non-vascular tissues are problems. As a result, cognitive abilities, skeletal deformities, or visual acuity are not improved. Some sources report that these symptoms may possibly be stabilized but not restored [8]. Thus, there is currently no treatment for neuronopathic types of MPS.

It is also worth noting the problem of MPS types I and II, as those types may (but not necessarily) have a neurodegenerative component. This depends largely on the type of mutation and, thus, the residual activity of the enzyme or its complete absence. However, there may be many more factors affecting enzyme activity, and these may include the efficiency of individual GAG synthesis, oxidative stress, endoplasmic reticulum stress, disruption of cell energetics, infection, defects in autophagy and lysosome biogenesis, disruption of calcium homeostasis, and many others [9]. 

Both treatment and diagnosis of neurological symptoms in MPS patients are difficult due to the lack of reaching the central nervous system (CNS) with current therapies and the lack of markers of neurodegeneration. Thus, it seems reasonable to identify pathogenic disorders present in the neuronopathic types of the disease (severe forms of MPS I and II, all MPS III and MPS VII) and thus not present in types without a neurodegenerative component (mild forms of MPS I and II, all MPS IV, MPS VI, and MPS IX). This approach, in addition to understanding the mechanisms of neurodegeneration itself, could lead to the designation of new therapeutic targets and diagnostic markers for neurodegeneration in MPS or to monitoring the effectiveness of the therapies used.

Organelle dysfunction is strongly associated with neurodegenerative disorders such as Alzheimer’s disease (AD) and Parkinson’s disease (PD). High mitochondrial dysfunction, consisting of impaired mitochondrial bioenergetics, impaired respiratory chain, impaired mitochondrial gene expression, mitophagy dysregulation, altered mitochondrial DNA levels, and increased oxidative stress, has already been observed for some time in AD and PD patients [10,11]. Changes in other organelles such as the endoplasmic reticulum, lysosomes, endosomes, or Golgi apparatus have also been correlated with neurodegenerative diseases [12,13,14,15]. Recent data indicated that the levels of organelle-related transcripts were disrupted and the number and morphology of individual cellular organelles were also altered in MPS [16,17,18,19]. In addition to other processes whose performance is disrupted [1,16,20,21,22,23,24,25,26,27,28,29,30], changes in the morphology of cellular organelles or changes in the expression levels of genes related to organelle function or structure may provide a marker for differentiating the course of MPS into those with or without a neurodegenerative component or, in the future, provide a therapeutic target for currently untreatable neurological types of the disease.

Therefore, the aim of this work was to identify genes related to the function or structure of cellular organelles that show changes in expression levels in neuronopathic types of MPS compared to healthy cells, while not showing these changes in non-neuronopathic types of MPS. In addition, the data identified in this work were compared with data on similar changes in other neurodegenerative diseases.

## 2. Materials and Methods

### 2.1. Cell Lines and Cell Cultures

Fibroblast lines taken from patients with neuronopathic (MPS I, MPS II, MPS III, and MPS VII) and non-neuronopathic (MPS IV, MPS VI, and MPS IX) types of MPS and purchased from the Coriell Institute were used for the experiments (Table 2). The HDFa (Human Dermal Fibroblast, adult) line was used as wild-type (healthy and control) cells. Cells were cultured under standard conditions at 37 °C, 95% humidity, in an atmosphere saturated with 5% CO_2_, in DMEM medium supplemented with 10% Fetal Bovine Serum (FBS), and in the presence of antibiotics.

### 2.2. Transcriptomic Analyses

#### 2.2.1. RNA Isolation and Purification

Fibroblasts (5 × 10^5^ cells) were passaged onto 10-centimeter-diameter plates and left overnight. To inactivate RNAases, cells were lysed with a solution containing guanidinium isothiocyanate and β-mercaptoethanol and then homogenized using a QIAshredder column. Subsequently, RNA was extracted using the RNeasy Mini kit (Qiagen, Hilden, Germany) and treated with Turbo DNase (Life Technologies, Life Technologies, Carlsbad, CA, USA) according to the manufacturer’s instructions. The quality of the isolated RNA samples was tested using Nano Chips RNA (Agilent Technologies, Santa Clara, CA, USA) on an Agilent 2100 Bioanalyzer system. RNA from each cell line was isolated in 4 independent replicates (4 independent biological repeats).

#### 2.2.2. RNA-Seq Analyses

The mRNA libraries were generated with the Illumina TruSeq Stranded mRNA Library Prep Kit. The cDNA libraries were sequenced on a HiSeq4000 (Illumina, San Diego, CA, USA). The following sequencing parameters were obtained: PE150 (150 bp paired-end) and a minimum of 40 million (40 M) of raw reads, which gave a minimum of 12 Gb of raw data per sample. Quality assessment was carried out by FastQC version v0.11.7. Raw readings were mapped to the GRCh38 human reference genome from the Ensembl database using the Hisat2 v. 2.1.0 program. To calculate the expression level of the transcripts, the Cuffquant and Cuffmerge programs in version 2.2.1 and the GTF Homo_sapiens.GRCh38.94.gtf file from the Ensembl database (https://www.ensembl.org/index.html, as of 19 February 2019) were used. The Cuffmerge program was started with the library-norm-method classic-fpkm parameter, normalizing the expression values by means of the FPKM algorithm. Transcript annotation and classification were performed using the BioMart interface for the Ensembl gene database (https://www.ensembl.org/info/data/biomart/index.html, as of 19 February 2019). RNA-seq data were deposited in the NCBI Sequence Read Archive (SRA) database: PRJNA562649.

#### 2.2.3. Statistical Analysis

Statistical analysis was performed using R v3.4.3 software using one-way analysis of variance (ANOVA) on log_2_(1 + x) values, which have a continuous normal distribution, and post hoc Student’s *t*-test with Bonferroni correction, as well as the Benjamini-Hochberg method for analyzing statistical significance between two groups and false discovery rate (FDR), respectively. Transcript classification was performed using the Ensembl database (BioMart interface) (https://www.ensembl.org/info/data/biomart/index.html, as of 19 February 2019). The procedure for RNA isolation and transcriptomic analysis using the RNA-seq technique has been described previously [23,31].

### 2.3. Electron Microscopy

Cells at 2 × 10^5^ were passaged into 12-well plates. The next day, cells were washed with PBS buffer, fixed with 2.5% glutaraldehyde, followed by 1% osmium tetroxide and 1% potassium hexacyanoferrate, and then dehydrated with ethanol. In the next step, cells were embedded in Epon 812 resin (Fluka Chemie GmbH, Buchs, Switzerland) and stained with lead citrate and uranyl acetate. Microscopic analyses were carried out using a Philips CM100 microscope with Olympus Soft Imaging Solution 1.4. software. Electron micrographs were used to evaluate the morphology of Golgi apparatuses. At least 100 cells were included in each case. The fragmentation features of Golgi apparatuses were compared between control cells and MPS cells, taking into account the division into neuronopathic and non-neuronopathic MPS types/subtypes.

### 2.4. Fluorescence Microscopy

Cells at 5 × 10^4^ were passaged on coverslips in 12-well plates. They were then fixed with 2% paraformaldehyde in PBS for 15 min, and sequentially in 0.1% Triton X-100 in PBS for 15 min. In the next step, the cells were washed 5 times with PBS. Golgi apparatuses were stained with CellLight™ Golgi-GFP BacMam 2.0 (#C10592, Invitrogen, Waltham, MA, USA) according to the manufacturer’s instructions. Cover slips were mounted on basal slides using ProLong™ Gold Antifade Mountant with DAPI closure medium (#P36935, Invitrogen). Microscopic analyses were carried out using a Leica DM4000 B fluorescence microscope (Leica Microsystems, Mannheim, Germany).

## 3. Results

Since disorders of the functions or structures of cellular organelles have increasingly been shown to be components of the pathogenesis of neurodegenerative diseases, this work set out to see if the phenomenon of disrupted organelle-related gene expression also occurs in neuronopathic types of MPS.

### 3.1. Transcriptomic Analyses 

Transcriptomic studies indicating abnormal expression of genes whose products are involved in the functions or structures of cell organelles were carried out by RNA-seq technique with a model of fibroblasts taken from patients with neuronopathic (MPS I, II, IIIA, IIIB, IIIC, IIID, and VII) and non-neuronopathic types/subtypes of MPS (MPS IVA, IVB, VI, and IX), as well as with control cells (HDFa, or Human Dermal Fibroblasts, adults). On this basis, transcripts were selected whose expression levels are altered in neuronopathic types of MPS but are not altered in non-neuronopathic types relative to control cells and whose products are involved in organelle function or structure; the following organelles were taken into consideration: Golgi apparatus (GO:0005794); mitochondrion (GO:0005739); ribosome (GO:0005840); nucleus (GO:0005634); cytoskeleton (GO:0005856); endoplasmic reticulum (GO:0005783); and vacuole (GO:0005773) (selection was made based on the Ensembl database as of 1 August 2023).

**Figure 1:**
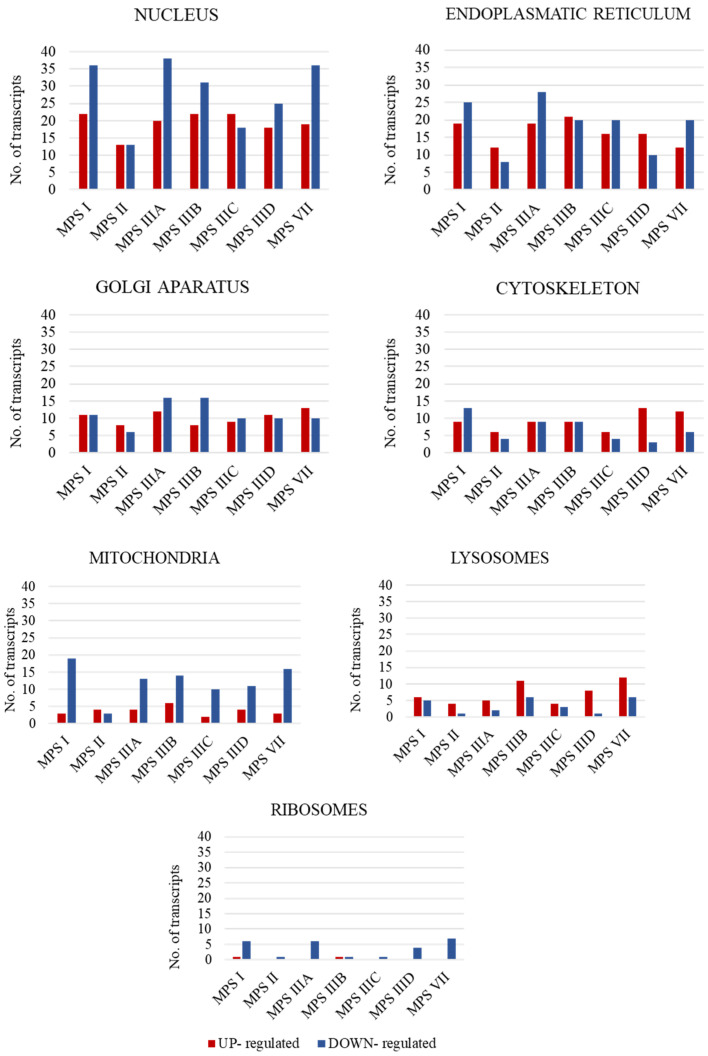
Number of statistically significant up- and down-regulated transcripts included in organelle-related GO terms, which are Golgi apparatus (GO:0005794); mitochondrion (GO:0005739); ribosome (GO: 0005840); nucleus (GO:0005634); cytoskeleton (GO:0005856); endoplasmic reticulum (GO:0005783); and vacuole (GO:0005773), in neuronopathic MPS types/subtypes relative to control cells (HDFa). Abnormal expression levels of these transcripts were not observed in non-neuronopathic MPS types/subtypes.

In the first stage of the study, the number of transcripts involved in the above-mentioned processes was determined. A large number of transcripts whose expression is disrupted only in neuronopathic types/subtypes of MPS (while not being altered in non-neuronopathic types) relative to control cells were identified for the cell nucleus (142 transcripts), endoplasmic reticulum (ER) (91 transcripts), and Golgi apparatus (57 transcripts), with down-regulated transcripts accounting for the vast majority (Figure 1).

In order to identify specific transcripts whose expression was disrupted in the greatest number of neuronopathic types/subtypes of the disease, those transcripts that appear in at least four neuronopathic types/subtypes of MPS were selected. The results of this selection by specific organelles are shown in Table 3.

Some of the selected transcripts appear frequently in different neuronopathic types/subtypes of the disease in question. Expression of the *ARL6IP6* gene, encoding ADP ribosylation factor like GTPase-interacting protein 6, was reduced in as many as six neuronopathic types/subtypes of MPS (out of seven tested). The ARL6IP6 protein is localized in the nuclear envelope and RE. It has the ability to shape high-curvature ER tubules, acts as a regulator of intracellular transport pathways in the ER membrane, and interacts with many proteins involved in membrane vesicle formation, also showing anti-apoptotic effects [32,33,34]. Among the ER-related genes selected were two more genes whose expression is up-regulated in five neuronopathic types/subtypes. These include *RPN2* and *PDIA3*. The *RPN2* gene encodes a highly conserved ribophorin II glycoprotein found in the membrane of the murine ER. The involvement of this glycoprotein has been confirmed by processes mediating the translocation of secretory proteins and maintaining the specificity of the murine ER, or N-glycosylating proteins. In contrast, *PDIA3* encodes the protein disulfide isomerase A3, which is a thioloxidoreductase found primarily in the RE endoplasm in the cytosol or cell nucleus [35]. The product of this gene, the PDIA3 protein, is responsible for regulating the folding of newly synthesized glycoproteins and promoting the re-folding of misfolded proteins. In addition, it protects the cell from entering the pathway of ER stress-induced apoptosis and interacts with lectin-binding chaperone proteins inside this organelle [36]. The *GOLGA2* gene, which encodes one of the golgins, a family of proteins located in the Golgi apparatus, was also within the scope of our interest. This protein plays an important role in the cisternae arrangement of the Golgi apparatus; vesicular transport interactions between the Golgi apparatus and microtubules are important for the reorganization of the Golgi apparatus after its fragmentation during mitosis [37].

Genes related to mitochondrial function and structure also often appeared on the list of selected genes. One of them is *MINOS1*, whose product is a protein of the inner mitochondrial membrane that affects its organization. In the MINOS1/Mio10 complex, it is responsible for maintaining the normal structure of mitochondria [38,39]. Another example is the *VASN* gene, which is associated with both mitochondria and lysosomes. The product of this gene is a type I trans-membrane glycoprotein that protects cells from apoptosis by regulating the activity of the mitochondrial antioxidant thioredoxin-2 [40,41]. 

**Figure 2:**
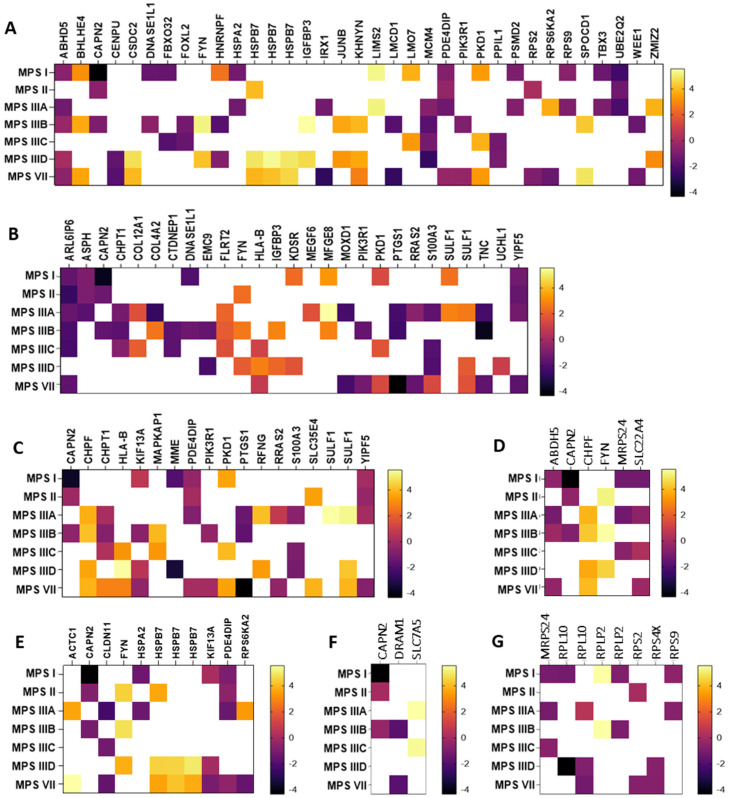
Heat maps presenting transcripts related to the nucleus (**A**), endoplasmic reticulum (**B**), Golgi apparatus (**C**), mitochondria (**D**), cytoskeleton (**E**), lysosome (**F**), and ribosomes (**G**) whose expression undergoes particularly high (log_2_FC > 1.5 or log_2_FC < −1.5) changes in neuronopathic MPS types/subtypes relative to control cells (HDFa). Abnormal expression levels of these transcripts were not observed in non-neuronopathic MPS types/subtypes.

In the next step, genes whose expression undergoes particularly high (log_2_FC > 1.5 or log_2_FC < −1.5) changes in expression in neuronopathic types/subtypes of MPS (while not undergoing changes in non-neuronopathic types) relative to healthy cells were selected. The selected transcripts are shown in Figure 2 as heat maps and in Supplementary Table S1 with the exact values of the fold change (log_2_FC).

Among the transcripts selected, there were both up-regulated (e.g., *MFGE8*, *HSPB7*, *SULF1*) and down-regulated (e.g., *ABHD5*, *PDE4DIP*, *YIPF5*, *CLDN11*). The *MFGE8* gene encodes milk fat globule-epidermal growth factor VIII, which maintains cellular homeostasis by alleviating ER stress [42]. Its role in phagocytosis of apoptotic cells, anti-inflammatory reactions, and tissue regeneration has also been reported [43]. Another example is the *HSPB7* gene up-regulated in MPS II, IIID, and VII, which encodes one of the most common small chaperone proteins located in the cell nucleus or cytoplasm. Recent reports indicate its major contribution to protection against neurodegeneration caused by protein aggregation in nerve cells [42]. Also associated with protein aggregates is the product of the *SULF1* gene synthesized in the Golgi apparatus, a surface sulfatase that removes specific 6-O-sulfate groups from heparan sulfate proteoglycans, thanks to which it can influence the modulation of various signaling pathways. Interest in this protein increased when its frequent occurrence in intracellular and extracellular protein aggregates was found. Studies indicated that heparan sulfate can interact with soluble proteins in different ways, causing them to fold abnormally into insoluble fibrils or aggregates [43].

An example of a severely down-regulated gene in MPS I, II, IIIA, and VII is *PDE4DIP*, encoding phosphodiesterase 4D interacting protein (PDE4DIP), a protein involved in the regulation of intracellular cAMP concentration [44,45]. PDE4DIP interacts with other proteins in cis-Golgi networks, making it an important component of ER-to-Golgi trafficking [46]. Another example of a gene that is particularly down-regulated in MPS I, IIIA, IIIB, IIID, and VII is *ABHD5*, which encodes Alpha/beta hydrolase domain-containing protein 5 (ABDH5 or CGI-58). This highly conserved protein is involved in regulating lipid metabolism and lipid droplet dynamics by activating the triglyceride hydrolase ATGL and other hydrolases [46,47,48]. The YIPF5 protein (a Yip1 domain family member 5), a product of the *YIPF5* gene, is involved in ER-to-Golgi transport.(. It is located in the ER and Golgi apparatus, as well as in vesicle transporters and down-regulated in some neuronopathic types/subtypes of MPS (I, II, IIIA, and VII). YIPF5-containing protein complexes are thought to play a key role in the transport of COPII-coated vesicles from the ER to the cis-Golgi and vesicle fusion to the Golgi apparatus [47]. The last gene to look at would be *CLDN11*, whose protein product (claudin 11) is a major component of central nervous system myelin and plays an important role in regulating oligodendrocyte proliferation and migration. Claudin 11 ensures rapid nerve conduction, mainly in myelinated axons of small diameter. Mice lacking claudin 11 have been shown to have preserved myelin and axonal architecture, but as much as 60% decreased in conduction. They are also characterized by an increased action potential threshold, severely slowed conduction velocities, and a large juxtaparanode potassium ion (K^+^) as a result of changes in the biophysical properties of myelin [48].

### 3.2. Electron and Fluorescent Microscopy

**Figure 3:**
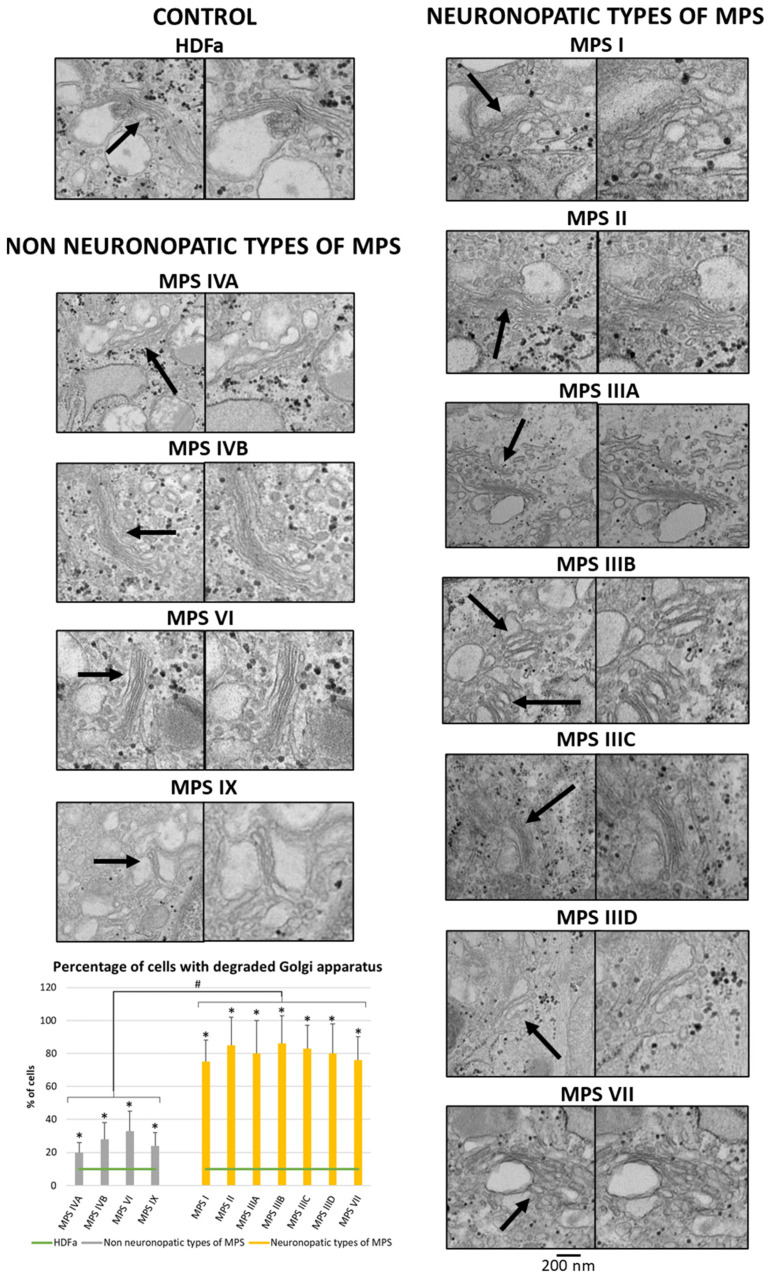
Morphology of the Golgi apparatus in neuronopathic and non-neuronopathic types/subtypes of MPS relative to control cells (HDFa) as studied by electron microscopy. The analysis of the percentage of fragmented Golgi apparatus structures was performed using at least 100 electron micrographs. Mean values ± SD are presented, with (*) representing statistically significant (*p* < 0.05) differences relative to the HDFa control and (#) representing statistically significant (*p* < 0.05) differences between neuronopathic and non-neuronopathic types/subtypes of MPS. Arrows indicate changes in Gogli apparatus morphology.

**Figure 4:**
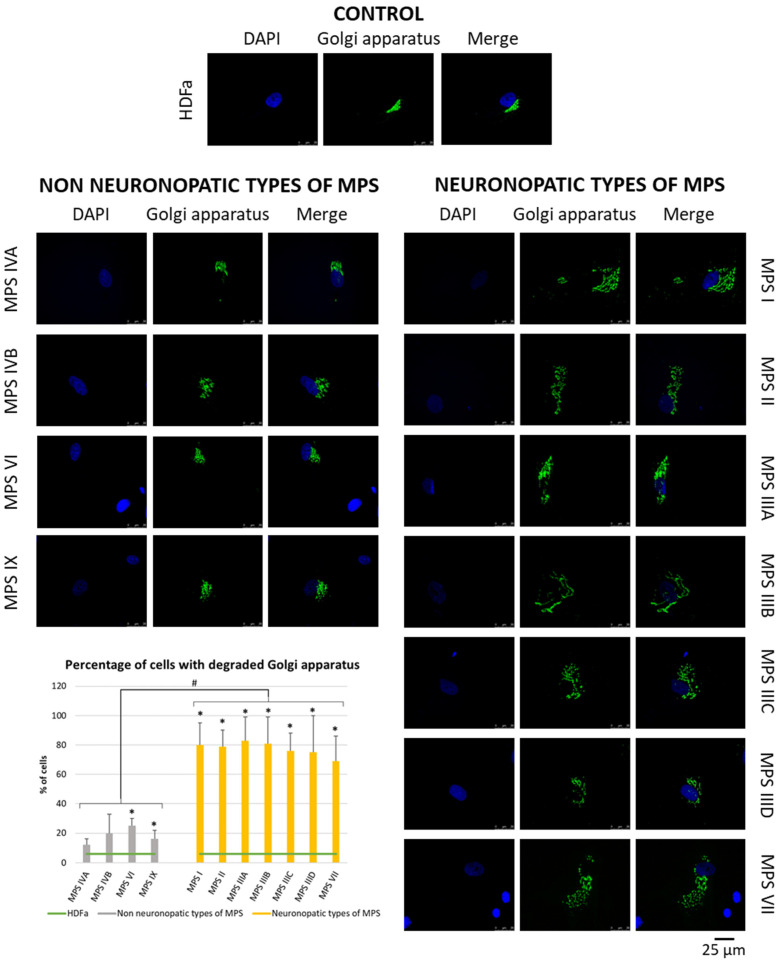
Morphology of the Golgi apparatus in neuronopathic and non-neuronopathic types/subtypes of MPS relative to control cells (HDFa) as studied by fluorescent microscopy using the fluorescent dye CellLight™ Golgi-GFP BacMam 2.0. The analysis of the percentage of fragmented Golgi apparatus structures was performed using at least 30 images. Mean values ± SD are presented, with (*) representing statistically significant (*p* < 0.05) differences relative to the HDFa control and (#) representing statistically significant (*p* < 0.05) differences between neuronopathic and non-neuronopathic types/subtypes of MPS.

Since many of the genes whose expression is disrupted in neuronopathic types/subtypes of MPS are related to the function or morphology of the Golgi apparatus and fragmentation of the Golgi apparatus is observed in many different neurodegenerative diseases, we decided to check whether this phenomenon is also present in those types/subtypes of MPS that are characterized by CNS symptoms. Visualization of the Golgi apparatus by electron microscopy and fluorescence microscopy indicated an increase in the number of components of the Golgi apparatus in neuronopathic types/subtypes of MPS compared to control cells and cells taken from patients with non-neuronopathic types/subtypes of MPS (Figure 3 and Figure 4, respectively). This fragmentation affects an average of 20% of the Golgi apparatus structures in MPS IV, VI, and IX, and as much as 80% of the Golgi apparatus structures in MPS I, II, III, and VII (Figure 4).

## 4. Discussion

The study of the mechanisms of neurodegeneration is of great interest to neuroscience specialists. Some data report that a component of the degeneration of cells in the nervous system is related to the function or structure of certain cellular organelles. This includes, for example, mitochondria, lysosomes, the cell nucleus, and the Golgi apparatus.

Studies on one of the most common neurological diseases, AD, have pointed to changes in the activities of enzymes involved in oxidative phosphorylation, oxidative damage, and mitochondrial binding of beta-amyloid and its precursor. Many mutations in genes encoding mitochondrial proteins in black matter neurons have been discovered in PD patients. Also noted in amyotrophic lateral sclerosis (ALS) are abnormalities in the efficiency of mitochondrial respiratory chain enzymes and mitochondrial programmed cell death proteins [49]. Mutations in genes encoding lysosomal proteins have been described not only in AD and ALS but also in frontotemporal dementia (FTD) and in lysosomal storage diseases (LSD) [15]. Lo and Zeng identified defective lysosomal acidification as an early indicator of neurodegeneration due to the onset of these disorders even before neurodegeneration occurs [50]. Fragmentation of the Golgi apparatus, as well as disturbances in the metabolism of proteins involved in maintaining its structure, have been described in AD, ALS, and Creutzfeldt-Jacob disease. Moreover, these events occur before clinical signs and other pathological manifestations become apparent [13,51,52]. 

Disorders of ER structure and function have also been identified in AD, PD, and prion diseases. On top of this, abnormalities of ER-phagy or RE degradation by lysosomes have been observed in AD, PD, Niemann–Pick type C, and schizophrenia [12,53]. Gupta and his team also identified large changes in the expression of genes encoding nuclear transcription factors by analyzing data from 300,000 patients with 30 different diseases of aging [54]. Some researchers emphasize that changes occurring within the structure of the aforementioned organelles or changes in the expression of genes related to their function occur even before the onset of the neurodegenerative process, pointing to these changes as possible markers of neuronal death.

Abnormalities in cellular organelle morphology and changes in the expression of organelle-related genes were also noted in MPS [16]. However, such abnormalities have never been considered markers to differentiate the neurodegenerative component of MPS. Table 4 compares the prevalence of abnormalities of the listed cell organelles in neuronopathic types/subtypes of MPS and other neurodegenerative diseases.

In this work, we identified genes related to the functions or structures of cellular organelles that showed changes in expression levels in neuronopathic types of MPS compared to healthy cells, while not showing these changes in non-neuronopathic types of MPS. Most such genes were identified as being characteristic of the cell nucleus, endoplasmic reticulum, and Golgi apparatus (Figure 1). Identification of these transcripts made it possible to select those that recur most frequently among the different neuronopathic MPS types/subtypes, or those whose changes in expression levels were particularly high (Table 3, Figure 2).

There is evidence that mutations or abnormal expression of the genes presented in this paper are associated with CNS symptoms. Mutations of the *ARL6IP6* gene, undergoing reduced expression in almost all neurodegenerative types/subtypes of MPS, have been found in many other neurodegenerative diseases. Neurological disorders such as abnormal gait, locomotor activation, limb grasping, and abnormal behavior have been indicated in mice with knockout of this gene [55]. *ARL6IP6* loss-of-function mutations have also been identified as a possible cause of cutis marmorata telangiectatica congenita, presenting with major dysmorphism, developmental delay, transient ischemic attacks, and cerebral vascular malformations. Additionally, they are associated with increased susceptibility to ischemic stroke [55,56] and the occurrence of disorders such as spastic paraplegia, diffuse sensorimotor polyneuropathy, and acromutilation [55,57]. Other studies have also indicated changes associated with the *PDIA3* gene in neurological diseases. Analysis performed as part of this work indicated that expression of this gene was elevated in neuronopathic types/subtypes of MPS. Expression levels of this gene were also elevated in the tissues of patients and mouse models affected by prion diseases, or ALS. *PDIA3* has been proposed as a possible risk factor and marker for the development of ALS [58]. Similarly, the *RPN2* gene encoding ribophorin II has recently been linked to AD. Studies on the mRNA level of the *RPN2* gene in the frontal or intraparietal cortex of AD patients indicated that it was reduced relative to controls [59]. Ribophorin II is a component protein of the subunits of STT3 complexes that catalyze N-glycosylation of emerging or misfolded proteins [60]. Mutations in genes encoding components of the aforementioned complexes cause congenital glycosylation disorders with impaired brain function [61]. 

Increased expression levels of the *MFGE8* gene in MPS cells are consistent with other neurodegenerative disease findings involving this gene. A beneficial role for MFGE8 has been demonstrated in stroke, neurodegenerative diseases (AD and PD), and traumatic brain injury. In stroke, MFGE8 promotes the proliferation of neural stem cells and their migration toward ischemic brain tissues [62]. Many studies point to an important role of MFGE8 in the process of neurogenesis, as its high levels reduce neuronal cell death through reduced expression of cleaved caspase-3 and IL-1β. This would suggest that the up-regulation of this gene’s expression level is a defense mechanism of the nervous system against neuronal cell death. The MFGE8 protein also has the ability to inhibit the production of pro-inflammatory cytokines or down-regulate the expression of apoptotic protein genes in models of cerebral ischemia and subarachnoid hemorrhage [62]. The need to study MFGE8 signaling pathways as a possible drug candidate for the bedside management of neurodegenerative diseases has been highlighted [62]. The *HSPB7* gene, encoding one of the molecular chaperones whose expression is up-regulated in MPS II, IIID, and VII, also appears to have a protective role in neurodegeneration. Studies of its role in diseases associated with polyQ expansion have indicated that it inhibits polyQ aggregation and prevents polyQ-induced toxicity [63]. Moreover, it was found that *HSPB7* overexpression alone did not increase autophagy, one of the mechanisms that may be responsible for the degradation of misfolded proteins. Nevertheless, in *ATG5*^−/−^ cells characterized by defective autophagy, HSPB7 activity against aggregation was significantly reduced. It was concluded that this chaperon prevents polyQ protein toxicity by a mechanism that requires active autophagy machinery [63]. Transcriptomic analyses also indicated up-regulation of *HSPB7* expression levels in dorsal root ganglia and sciatic nerve tissue in a mouse model of diabetic peripheral neuropathy [64]. The up-regulation of the *SULF1* gene, encoding sulfatase 1, observed in MPS I, IIIA, IID, and VII, may also be related to protein aggregation. The role of this sulfatase is to remove specific 6-O-sulfate groups from heparan-sulfate proteoglycans. Recent studies have indicated a role for 6-O sulfation in regulating the internalization of the tau protein, one of the hallmark proteins responsible for AD pathogenesis, in human central nervous system cell lines, iPS-derived neurons, and mouse brain slice cultures [65]. 

The down-regulation of *ABHD5* expression demonstrated in this work in MPS I, IIIA, IIIB, IIID, and VII was also observed in other CNS disorders. Mutations of this gene have been observed in patients with neutral lipid storage disease with neurological disorders [66] as well as Chanarin-Dorfman syndrome [67]. In both of these conditions, both somatic and neuronal symptoms appear. In addition to hepatic steatosis, skeletal myopathy, cardiomyopathy, and growth retardation, there are bilateral cataracts, ataxia, bilateral sensorineural hearing loss, and intellectual disability [67,68]. PDE4DIP, encoded by the gene of the same name, also appears to be an interesting protein. Its expression level is reduced in MPS I, II, IIIA, and VII. Deletion of a fragment of this gene resulted in behaviors characteristic of autism spectrum disorders, while duplication led to symptoms of psychosis/schizophrenia [69]. Bioinformatic analyses indicated that elevated *PDE4DIP* expression co-occurs with dementia in the course of neurodegenerative diseases such as AD, vascular dementia, frontotemporal dementia, or major depressive disorder [70,71]. A similar situation could be observed with reduced expression levels of *YIPF5* in MPS I, II, IIIA, and VII. Homozygous mutations of this gene have been observed as a genetic cause of congenital microcephaly syndrome, epilepsy, and neonatal/early-onset diabetes. In vitro studies indicated that loss of YIPF5 function resulted in proinsulin retention in the ER, marked ER stress, and β-cell failure. Partial silencing of this gene or the introduction of a mutation increased the sensitivity of β cells to ER stress-induced apoptosis [72]. The last example is *CLDN11*, which encodes claudin-11, which is a component of the myelin sheath of nerve cells [73]. *CLDN11* knockout mice are characterized by impaired auditory processing and reduced anxiety/avoidance. Importantly, these behaviors are associated with increased transmission time along myelinated fibers as well as imbalances of the neurotransmitters glutamate and GABA in the auditory brainstem and amygdala [74]. Abnormal expression of the *CLDN11* gene has also been reported in hypomyelinating leukodystrophy. Riedhammer et al. identified the *CLDN11* mutation in three patients suffering from this disease. The patients developed spastic movement disorder, expressive speech disorder, and eye abnormalities, including hypermetropia, and an MRI scan showed myelin deficits [73]. All of the genes discussed above, whose expression was altered in MPS, are included in a summary Table 5, indicating the role and function of the proteins they encode as well as the conditions associated with modulation of the expression of a particular gene.

In addition, since many of the genes indicated in the transcriptomic analyses relate to the function or morphology of the Golgi apparatus, it was decided to visualize it using electron and fluorescence microscopy. This analysis indicated fragmentation of this organelle in neuronopathic MPS types/subtypes to a greater extent than in non-neuronopathic MPS types/subtypes (Figure 3 and Figure 4). This is consistent with other literature data indicating that fragmentation and dispersion of the Golgi apparatus precede neuronal cell death. Nakagomi et al. indicated that pharmacological intervention or overproduction of the C-terminal fragment of Grasp65, a protein associated with the Golgi apparatus, inhibited fragmentation and reduced or delayed neuronal death. In addition, inhibition of pathways leading to cell death reduced fragmentation of the Golgi apparatus. The authors indicated that the Golgi apparatus may be a sensor of signals resulting in cell death [75]. Martínez-Menárguez and his team also pointed out that in many neurodegenerative diseases, such as AD, PD, or ALS, the Golgi apparatus did not take the form of a classical ribbon but was usually divided into isolated elements, which occurred as a very early event before clinical symptoms became apparent. However, it is not known whether this phenomenon is caused by mechanisms related to cell death or, conversely, initiates apoptosis [13]. Conducting studies focusing on the search for early pathological events occurring in the nerve cells of people affected by neurodegenerative diseases, Haukedal et al. identified Golgi fragmentation as one of the earliest phenotypes of AD, occurring even before the phenomena of increased Aβ secretion and tau hyperphosphorylation, as well as mitochondrial and synaptic deficits [76]. 

Turning to MPS, Golgi apparatus-related abnormalities in fibroblasts taken from MPS patients have already been indicated. Changes in the number of Golgi apparatus, which was increased, especially in MPS II, IIIA, IIIB, and IVB, were noted [16]. Detailed studies on the structure of the Golgi apparatus were performed on a mouse model of mucopolysaccharidosis type IIIB. An accumulation of intracellular storage vesicles bearing GM130 (GOLGA2), a Golgi matrix protein that mediates vesicle binding in both pre- and cis-Golgi compartments, was observed. An alteration of Golgi ribbon architecture, which comprised a distended cisterna connected to LAMP1-positive storage vesicles, was also noted. It was pointed out that the accumulation of vesicles and disorganization of the Golgi apparatus caused by changes in the expression of the *GM130* gene can cause neuronal dysfunction and death [77]. Our analysis identified genes that undergo impaired expression in neuronopathic types/subtypes of MPS while not undergoing changes in expression in non-neuronopathic types/subtypes and may be related to the observed changes in the structure of the Golgi apparatus. These include the already-mentioned *GM130* (*GOLGA2*) and *PDE4DIP* genes. *GOLGA2*, encoding golgin A2, undergoes increased expression in MPS I, IIIA, IIIB, IIIC, IIID, and IX. The studies cited above identified this gene as one of the key genes for the adoption of normal structure by the Golgi apparatus in a mouse model of MPS IIIB. These data are consistent with the data indicated in this paper on increased expression of the gene encoding this protein not only in MPS IIIB but also in other MPS types/subtypes. Shamseldin et al. also described a patient with a homozygous *GOLGA2* mutation with a neuromuscular disorder characterized by developmental delay, seizures, progressive microcephaly, and muscular dystrophy, while deletion of this gene in the striped danio also caused severe skeletal muscle disorganization and microcephaly [78]. Another gene that may be related to changes in the structure of the Golgi apparatus is the *PDE4DIP* gene, which encodes a phosphodiesterase 4D-interacting protein that undergoes reduced expression in MPS I, II, IIIA, and VII, which has already been described above. It was demonstrated that PDE4DIP interacts with other proteins in cis-Golgi networks, which affects the stability of both PDE4DIP itself and other proteins [46]. Moreover, disruption of this gene expression not only impairs the efficiency of transport from the ER to the Golgi apparatus but also causes fragmentation of the Golgi apparatus [46]. Experiments on the role of oxidative stress in maintaining the proper structure of the Golgi apparatus have indicated that activation of co-cellular stress elevates intracellular Ca^2+^ and protein kinase Cα (PKCα) activity, which phosphorylates the Golgi stacking protein GRASP55, leading to fragmentation of the Golgi apparatus [79]. A similar situation has been described for AD. A β-amyloid-induced increase in cytosolic Ca^2+^ levels leads to activation of calpain, which in turn activates Cdk5 kinase, leading to phosphorylation of the GRASP65 protein [52,80,81]. Interestingly, recent studies indicated that disruption of the Golgi structure by knocking out GRASP55 and GRASP65 increases HS synthesis and decreases CS synthesis in cells, as well as changing the sulfation pattern and decreasing the secretion of both of these GAGs. Those studies provided evidence that a structural defect in the Golgi apparatus can significantly alter GAG synthesis and secretion [82].

Taking into account both the above-described abnormalities of gene expression related to the functions/structures of cellular organelles and the fragmentation of the Golgi apparatus in neurodegenerative diseases, it should not be surprising that symptoms occurring in the course of MPS I, II, III, and VII sometimes cause misdiagnosis, indicating either a neurological disease or a psychiatric disorder [83]. Escolar et al. compiled information on the use of drugs affecting behavior and mood in the treatment of MPS, which are typically used for psychiatric disorders. The data presented shows that the use of pharmacotherapy to treat sleep, mood, and other behavioral disorders affects patients with MPS to varying degrees, but the authors pointed to the benefits of using the described drugs as complementary therapy [84]. 

It may be surprising to use fibroblasts taken from patients to study neurodegenerative diseases. It is worth mentioning, however, that research conducted with a model of fibroblasts that are not nerve cells is nevertheless quite common. On the one hand, this is certainly a matter of the difficulty of collecting and culturing neurons. On the other hand, fibroblasts could be relatively easily obtained from patients and control individuals, making the research material both specific and homogeneous. The use of patient-derived cell lines is also advantageous relative to heavily modified cells, like neuronal cells differentiated under laboratory conditions, where potential off-target effects should be taken into consideration, the problem which is absent in fibroblasts obtained directly from affected individuals. It has already been documented that primary fibroblasts from patients with PD, AD, and spinal–cerebellar ataxia type 2 showed a distinct and unique mRNA expression pattern of key genes for neurodegeneration [85]. This approach was also supported by Olesen et al., who presented data on mitochondrial abnormalities from fibroblasts taken from AD, PD, HD, and ALS patients, and these changes turned out to be similar to those observed in the CNS. The authors pointed to fibroblasts as a reliable model for detecting early abnormalities because of their metabolic and biochemical links to neurons [86]. More importantly, with recent advances in single-cell RNA sequencing, the heterogeneity and diversity of fibroblasts within the CNS have been discovered. Based on their distinct anatomical localization in the meninges, perivascular space, and choroid plexus, as well as their molecular diversity, fibroblasts are said to play an important role in the CNS in both physiological and pathological states [87]. The usefulness of experiments with fibroblasts for studies on neurodegenerative diseases, like AD and other disorders, as well as on the functions of the CNS, was also highlighted recently [88,89,90,91,92]. Therefore, we believe that results obtained with fibroblast models can be reliable in studies on mechanisms of neurodegeneration, especially when gene expression patterns are analyzed.

## 5. Conclusions

In summary, the scope of this study was to compare the molecular features of neuronopathic types of MPS with those of other neurodegenerative diseases. Indeed, we demonstrated that, besides the fact that patients suffering from neuronopathic types/subtypes of MPS and other neurodegenerative diseases share many clinical similarities, there are also molecular similarities between these diseases. Changes in the expression of genes encoding organelle-related proteins and consequent disruption of the organelle structure or function itself are common factors in many entities associated with CNS damage. Here, we have pointed out that neuronopathic types/subtypes of MPS are also among such diseases. Genes whose expression is affected in neuronopathic MPS are often associated with the structures or functions of the cell nucleus, endoplasmic reticulum, or Golgi apparatus, and disruptions in the structures of these organelles could be confirmed. Among genes with changed expression levels in neuronopathic MPS types were up-regulated ones, like *PDIA3*, *GM130* (*GOLGA2*), and *MFGE8*, and down-regulated ones, like *ARL6IP6*, *ABHD5*, *PDE4DIP*, *YIPF5*, and *CLDN11*. Disorders of the described organelle-related genes and changes in the morphology of the Golgi apparatus were not noted in non-neuronopathic types/subtypes of this disorder. Taking into account that for neuronopathic types/subtypes of MPS there is currently no treatment, and in the case of MPS types I and II, the neurodegenerative component is, with the current state of knowledge, impossible to predict, we propose to consider the genes presented in this paper as candidates for markers of neurodegeneration in MPS. 

Limitations of this study include the use of fibroblasts (rather than neural cells) as models of neurodegenerative diseases and the employment of only one cell line per MPS type. However, it was indicated previously that fibroblasts could be useful models in neurodegenerative diseases, and peripheral markers are also usable when assessing neurological disorders. Moreover, since directions of dysregulation (either up-regulation or down-regulation) of specific genes were in most cases the same among all neurodegenerative MPS types, one might conclude that the results of analyses based on one line per MPS type are reliable. Nevertheless, the proposed hypothesis requires verification in the largest possible group of patients with neuronopathic and non-neuronopathic types of MPS, which may be difficult in rare diseases where the overall number of patients is small. However, only such studies would be able to indicate whether the proposal of abnormal expression of organelle-related genes/abnormal organelle morphology as markers of neurodegeneration in MPS would work in practice. These genes or the proteins they encode could also become potential targets for therapies under development for neurological disorders associated with the disease and markers of the effectiveness of the use of these therapies.

## Figures and Tables

**Table 1:** Characteristics of different types/subtypes of MPS.

MPS Type ^#^	Defective Gene	Defective Enzyme	Stored GAG(s) *
MPS I	*IDUA*	α-L-iduronidase	HS, DS
MPS II	*IDS*	2-iduronate sulfatase	
MPS IIIA	*SGSH*	N-sulfoglucosamine sulfhydrolase	HS
MPS IIIB	*NAGLU*	α-N-acetylglucosaminidase	
MPS IIIC	*HGSNAT*	Acetyl-CoA:α-glycosaminide acetyltransferas	
MPS IIID	*GNS*	N-acetylglucosamine 6-sulfatase	
MPS IIIE	*ARSG*	Arylusulfatase G	
MPS IVA	*GLANS*	N-Acetyloglucosaminase-6-sulfate sulfatase	C6S, KS
MPS IVB	*GLB1*	β-Galactosidase	KS
MPS VI	*ARSB*	Arylusulfatase B	DS, C4S
MPS VII	*GUSB*	N-acetylgalactosamine 4-sulfatase	HS, DS, CS
MPS IX	*HYAL1*	Hyaluronidase	HA
MPS X	*ARSK*	Arylusulfatase K	DS
MPS-PS	*VPS33A*	VPS33A	HS, DS

^#^ MPS IIIE is not included into MPS by some researches, as studies conducted with patients’ materials confirmed a decrease in the enzymatic activity, but not an increase in GAG levels. Moreover, the clinical picture did not resemble any of the MPS III subtypes, nor did it have the typical features of MPS. This gave rise to the diagnosis of atypical Usher’s disease in the patients. On the other hand, classical MPS IIIE with *ARSG* mutation and increased GAG levels was identified in the mouse model. MPS-plus syndrome (MPS-PS) is also questioned to be an MPS type, as despite GAG accumulation and symptoms typical for MPS, there is no decrease in the activity of lysosomal enzymes responsible for GAG degradation. * Abbreviations: CS, chondroitin sulfate; DS, dermatan sulfate; HA, hyaluronic acid; HS, heparan sulfate; KS, keratan sulfate.

**Table 2:** Characteristics of MPS cell lines used in the study.

MPS Type	Stored GAG(s) *	Defective Enzyme	Mutation Type	Cat. Number of the Cell Line **
MPS I	HS, DS	α-L-iduronidase	p.Trp402Ter/p.Trp402Te	GM00798
MPS II		2-iduronate sulfatase	p.His70ProfsTer29	GM13203
MPS IIIA	HS	N-sulfoglucosamine sulfhydrolase	p.Glu447Lys/p.Arg245His	GM00879
MPS IIIB		α-N-acetylglucosaminidase	p.Arg626Ter/p.Arg626Ter	GM00156
MPS IIIC		Acetyl-CoA:α-glycosaminide acetyltransferas	p.Gly262Arg/pArg509Asp	GM05157
MPS IIID		N-acetylglucosamine 6-sulfatase	p.Arg355Ter/p.Arg355Ter	GM05093
MPS IVA	KS, CS	N-acetylglucosamine- 6-sulfate sulfatase	p.Arg386Cys/p.Phe285Ter	GM00593
MPS IVB		β-galactosidase	p.Trp273Leu/p.Trp509Cys	GM03251
MPS VI	DS, C4S	N-acetylglucosamine- 4-sulfatase (arylsulfatase B)	Not determined	GM03722
MPS VII	HS, DS, CS	N-acetylgalactosamine 4-sulfatase	p.Trp627Cys/p.Arg356Ter	GM17494
MPS IX	HA	Hyaluronidase	p.Glu268Lys/c.37bp-del;14bp-ins at nt 1361	GM17494
Control (HDFa)	None	N/A	N/A	N/A

* Abbreviations: CS, chondroitin sulfate; DS, dermatan sulfate; HA, hyaluronic acid; HS, heparan sulfate; KS, keratan sulfate; N/A, not applicable. ** Cat. numbers are according to the cell line description at the Coriell Institute.

**Table 3:** List of transcripts whose expression is altered in at least four neuronopathic types of MPS.

Organellum	No. of Genes with Altered Expression in a Given Structure	Selected Genes	Number of MPS Types/Subtypes in Which Gene Expression Has Changed	Regulation of Expression vs. Control Cells	MPS Types/Subtypes in Which Altered Gene Expression Has Been Detected
Nucleus	142	*ARL6IP6*	6	↓	I, II, IIIA, IIIB, IIIC, VII
		*PDIA3*	5	↑	I, II, IIIA, IIIB, IIIC
		*RPN2*	5	↑	I, II, IIIA, IIIB, IIID
		*ABHD5*	4	↓	I, IIIA, IIIB, VII
		*DMWD*	4	↑	IIIB, IIIC, IIID, VII
		*MCM4*	4	↓	IIIA, IIIB, IIIC, IIID
		*PDE4DIP*	4	↓	I, II, IIIA, VII
		*SORBS3*	4	↑	I, IIIA, IIIB, IIID
		*TIGAR*	4	↓	I, IIIB, IIIC, VII
Endoplasmic reticulum	91	*ARL6IP6*	6	↓	I, II, IIIA, IIIB, IIIC, VII
		*RPN2*	5	↑	I, II, IIIA, IIIB, IIID
		*PDIA3*	5	↑	I, II, IIIA, IIIB, IIIC
		*BCAP29*	4	↓	I, IIIA, IIIB, IIIC
		*CPED1*	4	↓	I, IIIA, IIIB, VII
		*SDC2*	4	↓	I, IIIA, IIIC, VII
		*STS*	4	↓	IIIB, IIIC, IIID, VII
		*YIPF5*	4	↓	I, II, IIIA, VII
Golgi apparatus	57	*GOLGA2*	5	↑	I, IIIA, IIIB, IIIC, IIID
		*CHPF*	4	↑	IIIA, IIIB, IIID, VII
		*KIF13A*	4	↓	I, IIIB, IIID, VII
		*PDE4DIP*	4	↓	I, II, IIIA, VII
		*S100A3*	4	↓	IIIA, IIID, IIIC, VII
		*SDC2*	4	↓	I, IIIA, IIIC, VII
		*STS*	4	↓	IIIB, IIIC, IIID, VII
		*YIPF5*	4	↓	I, II, IIIA, VII
Mitochondrion	43	*MINOS1*	5	↓	I, IIIB, IIIC, IIID, VII
		*VASN*	5	↑	II, IIIA, IIIB, IIID, VII
		*ABHD5*	4	↓	I, IIIA, IIIB, VII
		*CHPF*	4	↑	IIIA, IIIB, IIID, VII
		*SLC22A4*	4	↓	I, IIIA, IIIC, VII
		*TIGAR*	4	↓	I, IIIB, IIIC, VII
Cytoskeleton	48	*KIF13A*	4	↓	I, IIIB, IIID, VII
		*PDE4DIP*	4	↓	I, II, IIIA, VII
		*SORBS3*	4	↑	I, IIIA, IIIB, IIID, VII
Lysosome	22	*VASN*	5	↑	II, IIIA, IIIB, IIID, VII
		*CCZ1*	4	↑(I, IIID, VII); ↓(IIIB)	I, IIIB, IIID, VII
		*SDC2*	4	↓	I, IIIA, IIIC, VII
		*STS*	4	↓	IIIB, IIIC, IIID, VII

Symbols: ↓, down-regulation of gene expression; ↑, up-regulation of gene expression.

**Table 4:** Abnormalities related to the function or morphology of cell organelles in MPS and other neurological disease/disturbances or mental disorders.

Neurological Disease/Disturbances *	Lysosome	Cytoskeleton	Nucleus	ER	Ribosomes	Golgi	Mitochondria
MPS I, II, III, VII	+	+	+	+	+	+	+
Alzheimer disease	+	+		+	+	+	+
Parkinson disease	+	+		+	+	+	+
ALS	+	+		+	+	+	+
SMA				+	+		+
Cognitive disturbances				+	+		+
Huntington’s disease	+				+		+
Stroke		+					+
Epilepsy		+		+	+		+
Friedreich’s ataxia							+
Tourette Syndrome							+
Williams Syndrome							+
Type I lissencephaly		+					
Intellectual disabilities		+					
FXTAS					+		
OPMD			+				
Intracerebral hemorrhage					+		+
Christianson syndrome	+						
Ischemia				+			
Mental Disorders *	**Lysosomes**	**Cytoskeleton**	**Nucleus**	**ER**	**Ribosomes**	**Golgi**	**Mitochondria**
ADHD							+
Schizophrenia		+	+	+	+		+
Mood Disorders		+		+			+
ASD	+	+	+	+	+	+	+
Bipolar disorder		+	+	+	+		+
Depression		+		+	+		+
Personality disorders							+
OCD							+
Rett Syndrome			+				
Frontal Dementia			+		+		
PTSD				+	+		
Addictions				+			
FTD	+				+		

* Abbreviations and symbols: ER, endoplasmic reticulum; ALS, amyotrophic lateral sclerosis; SMA, spinal muscular atrophy; FXATS, fragile X-associated tremor/ataxia syndrome; OPMD, oculopharyngeal muscular dystrophy; ADHD, attention deficit/hyperactivity disorder; ASD, autism spectrum disorder; OCD, obsessive-compulsive disorder; PTSD, post-traumatic stress disorder; FTD, frontotemporal dementia; +, the occurrence abnormalities in specific organelle or cellular structure.

**Table 5:** Characterization of genes whose expression is altered in MPS types/subtypes, their products, and related diseases.

Gene	Protein	Function	Localization	MPS Type	Regulation	Other Diseases
*ARL6IP6*	ADP ribosylation factor-like GTPase 6 interacting protein 6	high-curvature ER tubules, regulation of intracellular transport pathways, interaction with proteins involved in membrane vesicle formation	nuclear envelope, RE	I II IIIA IIIB IIIC VII	↓	cutis marmorata telangiectatica congenita, ischemic stroke, spastic paraplegia, diffuse sensorimotor polyneuropathy, acromutilation
*PDIA3*	protein disulfide isomerase A3	regulation of the folding of newly synthesized glycoproteins, promotion of the re-folding of misfolded proteins	nucleus, RE endoplasm, cytoplasm	I II IIIA IIIB IIIC	↑	prion disease, ALS
*RPN2*	ribophorin II glycoprotein	mediation of the translocation of secretory proteins, maintainance of the specificity of ER, N-glycosylation proteins	membrane of the ER	I, II IIIA IIIB IIID	↑	AD
*MFGE8*	milk fat globule- epidermalgrowth factor VIII	alleviation of ER stress, apoptotic cell phagocytosis, anti-inflammatory reactions, tissue regeneration	cytoplasm	I IIIA IIIB	↑	subarachnoid hemorrhage, cerebral ischemia (stroke), AD, PD, traumatic brain injury
*HSPB7*	heat shock protein family B (small) member 7	chaperone protein	nucleus, cytoplasm	II IIID VII	↑	poliQ-related diseases, diabetic peripheral neuropathy
*SULF1*	sulfatase 1	removal of specific 6-O-sulfate groups from heparan- sulfate proteoglycans	Golgi apparatus	I, IIIA IIID VII	↑	AD
*ABHD5*	alpha/beta hydrolase domain-containing protein 5	regulation of lipid metabolism and lipid droplet dynamics	cytoplasm, nucleus, mitochondria	I IIIA IIIB IIID VII	↓	lipid storage disease with neurological disorders, Chanarin- Dorfman syndrome
*PDE4DIP*	phosphodiesterase 4D-interacting protein	regulation of intracellular cAMP concentration, component of ER-to-Golgi trafficking, maintainance of the structure of Golgi apparatus	Golgi apparatus, centrosome	I II IIIA VII	↓	autism spectrum disorders, psychosis, schizophrenia, AD, vascular dementia, frontotemporal dementia, major depressive disorder
*YIPF5*	Yip1 domain family member 5	transport of COPII-coated vesicles from the ER to the cis-Golgi and vesicle fusion to the Golgi apparatus	ER, Golgi apparatus, vesicle transporters	I II IIIA VII	↓	congenital microcephaly syndrome, epilepsy, neonatal/early-onset diabetes
*CLDN11*	claudin-11	component of the myelin sheath of nerve cells, transmission along myelin fibers, maintainance of the balance of neurotransmitters	plasma membrane, cytoskeleton	IIIA IIIC VII	↓	hypomyelinating leukodystrophy

Abbreviations and symbols: ER, endoplasmic reticulum; AD, Alzheimer disease; PD, Parkinson disease; ALS, amyotrophic lateral sclerosis; ↓, down-regulation of gene expression; ↑, up-regulation of gene expression.

## Data Availability

RNA-seq raw data were deposited in the NCBI Sequence Read Archive (SRA) with accession No. PRJNA562649.

## Supplementary Materials

The following supporting information can be downloaded at: https://www.mdpi.com/article/10.3390/cimb46030169/s1. Table S1: Transcripts with severely impaired expression (log_2_FC < −1.5 or >1.5) in neuronopathic types/subtypes of MPS associated with organelles.
