# Supplementary material for: Cellular Organelle-Related Transcriptomic Profile Abnormalities in Neuronopathic Types of Mucopolysaccharidosis: A Comparison with Other Neurodegenerative Diseases
Source: Curr Issues Mol Biol. 2024 Mar 21;46(3):2678–700. doi: 10.3390/cimb46030169 (PMC10968730; doi:10.3390/cimb46030169)
Supplement: Supplementary file 1 [file cimb-46-00169-s001.zip › CIMB-Organella-supplementary-Table-S1.pdf]

Table S1. Transcripts with severely impaired expression ($log_2FC<-1.5$ or >1.5) in neuronopathic types/subtypes of MPS associated with organelles.

Structure or organellum	Transcripts with severely impaired expression ($log_2FC<-1.5$ or >1.5) in specific neuronopathic types/subtypes of MPS associated with organelles							
	**Transcript**	**MPS I**	**MPS II**	**MPS IIIA**	**MPS IIIB**	**MPS IIIC**	**MPS IIID**	**MPS VII**
Nucleus	*ABHD5*	-	-	-1.75	-	-	-	-
	BHLHE4	-	-	-	1.56	-	-	-
	CAPN2	-3.87	-	-	-1.58	-	-	-
	CENPU	-	-	-	-	-	-1.99	-2.2
	CSDC2	-	-	-	-	-	2.29	1.76
	DNASE1L1	-1.95	-	-	-	-	-	-
	FBXO32	-1.96	-	-	-	-2.21	-	-
	FOXL2	-	-	-	-	-1.73	-1.93	-
	FYN	-	-	-	2.05	-	1.89	-
	HNRNPF	-	-	-	-2.19	-	-	-
	HSPA2	-1.73	-	-1.75	-	-	-	-
	HSPB7	-	1.8	-	-	-	2.70	1.85
	HSPB7	-	-	-	-	-	2.38	1.72
	HSPB7	-	-	-	-	-	2.4	2.2
	IGFBP3	-	-	-	2.08	-	2.21	-
	IRX1	-	-	-1.74	-	-	-	-2.82
	JUNB	-	-	-	1.52	-	-	-
	KHNYN	-	-	-	1.75	-	1.55	-
	LIMS2	2.66	-	2.58	-	-	-	-
	LMCD1	-	-	-2.178	-	-	-	-2.75
	LMO7	1.65	-	-	-	-	-	-
	MCM4	-	-	-	-2.14	-1.69	-2.72	-
	PDE4DIP	-	-	-1.8	-	-	-	-
	PIK3R1	-	-	-1.52	-	-	-	-
	PKD1	-	-	-	-	1.67	-	-
	PPIL1	-	-	-	-	-1.73	-1.64	-
	PSMD2	-1.57	-	-	-	-	-	-
	RPS2	-	-	-	-	-	-	-
	RPS6KA2	-	-	1.59	-	-	-	-
	RPS9	-	-1.52	-	-	-	-	-1.88
	SPOCD1	-	-	-	1.96	-	-	2.39
	TBX3	-1.89	-	-1.56	-	-	-	-
	UBE2Q2	-2.55	-1.82	-2.37	-	-	-	-
	WEE1	-	-	-	-1.88	-	-	-1.77
	ZMIZ2	-	-	1.66	-	-	-	-
Endoplasmic reticulum	*ARL6IP6*	-1.57	-2.61	-1.47	-2.12	-2.19	-	-1.64
	ASPH	-	-	-1.79	-	-	-	-
	CAPN2	-3.87	-	-	-1.58	-	-	-
	CHPT1	-	-	-	-1.75	-	-	-
	COL12A1	-	-	1.53	-	1.91	-	-
	COL4A2	-	-	-2.63	2.51	-	-	-
	CTDNEP1	-	-	-	-1.73	-1.74	-	-
	DNASE1L1	-1.95	-	-	-	-	-	-

	EMC9	-	-	-	-1.54	-	-	-2.18
	FLRT2	-	-	2.10	1.9	1.54	-	-
	FYN	-	2.37	-	2.05	-	1.89	-
	HLA-B	-	-	-	-	-	2.08	-
	IGFBP3	-	-	-	2.08	-	2.21	-
	KDSR	2.03	-	-	-	-	1.64	-
	MEGF6	-	-	1.59	-	-	-	-
	MFGE8	3.03	-	5.05	2.82	-	-	-
	MOXD1	-	-	-2.25	-	-	-	-2.28
	PIK3R1	-	-	-	-1.52	-	-	-
	PKD1	-	-	-	-	1.67	-	-
	PTGS1	-	-	-2.22	-2.38	-	-	-4.32
	RRAS2	-	-	-	-	-	-	-1.52
	S100A3	-	-	-1.65	-	-1.88	-1.98	-1.65
	SULF1	2.04	-	2.80	-	-	-	-
	SULF1	-	-	2.63	-	-	-	-
	TNC	-	-	-2.25	-3.85	-	-	-1.63
	UCHL1	-	-	-	2.42	-	-	-
	YIPF5	-	-	-	-	-	-	-1.71
Golgi apparatus	*CAPN2*	-3.87	-	-	-1.58	-	-	-
	CHPF	-	-	1.52	1.75	-	-	1.58
	CHPT1	-	-	-	-1.75	-	-	-
	HLA-B	-	-	-	-	-	2.08	-
	KIF13A	-	-	-	-1.65	-	-	-1.54
	MAPKAP1	-	-	-	1.65	-	-	-
	MME	-2.61	-	-	-	-	-3.62	-
	PDE4DIP	-	-	-1.8	-	-	-	-
	PIK3R1	-	-	-	-1.52	-	-	-
	PKD1	-	-	-	-	1.67	-	-
	PTGS1	-	-	-2.22	-2.38	-	-	-4.32
	RFNG	-	-	1.74	-	-	-	-
	RRAS2	-	-	-	-	-	-	-1.52
	S100A3	-	-	-1.65	-	-1.88	-1.98	-1.65
	SLC35E4	-	-	-	-	-	-	1.58
	SULF1	2.04	2.80	-	-	-	-	-
	SULF1	-	2.63	-	-	-	1.85	1.62
	YIPF5	-	-	-	-	-	-	-1.71
Mitochondrion	*ABHD5*	-	-	-1.75	-	-	-	-
	CAPN2	-3.87	-	-	-1.58	-	-	-
	CHPF	-	-	1.52	1.76	-	-	1.58
	FYN	-	2.37	-	2.05	-	1.89	-
	MRPS24	-1.84	-	-1.85	-	-	-	-
	SLC22A4	-1.83	-	-	-	-	-	-
Cytoskeleton	*ACTC1*	-	-	1.74	-	-	-	3.15
	CAPN2	-3.87	-	-	-1.58	-	-	-
	CLDN11	-	-	-2.43	-	-1.64	-	-1.91
	FYN	-	2.37	-	2.05	-	1.89	
	HSPA2	-1.73	-	-1.75	-	-	-	-
	HSPB7	-	1.8	-	-	-	2.70	1.85

	HSPB7	-	-	-	-	-	2.38	1.72
	HSPB7	-	-	-	-	-	2.4	2.2
	KIF13A	-	-	-	-	-	-	-1.54
	RPS6KA2	-	-	1.59	-	-	-	-
	PDE4DIP	-	-	-1.8	-	-	-	-
Lysosome	*CAPN2*	-3.87	-1.36	-	-1.58	-	-	-
	DRAM1	-	-	-	-2.41	-	-	-2.4
	SLC7A5	-	-	1.82	-	1.73	-	-
Ribosome	*MRPS24*	-1.84	-	-1.85	-	-	-	-
	RPL10	-1.89	-	-	-	-	-4.61	-
	RPL10	-	-	-	-	-	-1.62	-1.94
	RPLP2	-1.66	-	-	-1.72	-	-	-
	RPLP2	3.80	-	-	3.81	-	-	-
	RPS2	-	-	-	-	-	-	-1.55
	RPS4X	-	-	-	-	-	-1.53	-
	RPS9	-	-	-	-	-	-1.55	-1.56
